# Meaningful Patient Engagement in Adolescent and Young Adult (AYA) Cancer Research: A Framework for Qualitative Studies

**DOI:** 10.3390/curroncol31040128

**Published:** 2024-03-22

**Authors:** Niki Oveisi, Vicki Cheng, Dani Taylor, Haydn Bechthold, Mikaela Barnes, Norman Jansen, Helen McTaggart-Cowan, Lori A. Brotto, Stuart Peacock, Gillian E. Hanley, Sharlene Gill, Meera Rayar, Amirrtha Srikanthan, Mary A. De Vera

**Affiliations:** 1 Faculty of Pharmaceutical Sciences, University of British Columbia, Vancouver, BC V6T 1Z3, Canada; niki.oveisi@ubc.ca (N.O.); vcheng1@student.ubc.ca (V.C.); 2 Collaboration for Outcomes Research and Evaluation, Vancouver, BC V6T 1Z3, Canada; 3 Patient Research Partner; 4 Registered Physiotherapist, Pelvic Health Provider, Vancouver, BC, Canada; 5 BC Cancer, Vancouver, BC V5Z 1M9, Canada; hcowan@bccrc.ca (H.M.-C.); speacock@bccrc.ca (S.P.); sgill@bccancer.bc.ca (S.G.); 6 Faculty of Health Sciences, Simon Fraser University, Burnaby, BC V5A 1S6, Canada; 7 Faculty of Medicine, University of British Columbia, Vancouver, BC V6T 1Z3, Canada; lori.brotto@ubc.ca (L.A.B.); gillian.hanley@ubc.ca (G.E.H.); meera.rayar@cw.bc.ca (M.R.); 8 Faculty of Medicine, University of Ottawa, Ottawa, ON K1H 8M5, Canada; asrikanthan@toh.ca; 9 Division of Medical Oncology, Department of Medicine, The Ottawa Hospital, Ottawa, ON K1H 8M5, Canada; 10 The Ottawa Hospital Research Institute, Ottawa, ON K1Y 4E9, Canada; 11 Centre for Health Evaluation and Outcome Sciences, Vancouver, BC V6Z 1Y6, Canada

**Keywords:** patient-centered research, sexual and reproductive health, cancer survivorship, adolescent and young adult, qualitative research

## Abstract

Over the last two decades, patient engagement in cancer research has evolved significantly, especially in addressing the unique challenges faced by adolescent and young adult (AYA) cancer populations. This paper introduces a framework for meaningful engagement with AYA cancer patient research partners, drawing insights from the “FUTURE” Study, a qualitative study that utilizes focus groups to explore the impact of cancer diagnosis and treatment on the sexual and reproductive health of AYA cancer patients in Canada. The framework’s development integrates insights from prior works and addresses challenges with patient engagement in research specific to AYA cancer populations. The framework is guided by overarching principles (safety, flexibility, and sensitivity) and includes considerations that apply across all phases of a research study (collaboration; iteration; communication; and equity, diversity, and inclusion) and tasks that apply to specific phases of a research study (developing, conducting, and translating the study). The proposed framework seeks to increase patient engagement in AYA cancer research beyond a supplementary aspect to an integral component for conducting research with impact on patients.

## 1. Introduction

The past two decades have seen growth in patient engagement in cancer research; that is, involving patients as partners across all phases of a study beyond the traditional roles as participants [1,2,3]. Indeed, with the complexity of cancer, its treatments, and significant short- and long-term impacts, involving patients in the design, conduct, and translation enriches cancer research with lived expertise. Various guiding documents have been developed to support and foster patient engagement in cancer research. In 2018, Deverka et al. developed a framework for patient engagement focusing on early phases of clinical trial concept development at the SWOG Cancer Research Network, the largest cancer clinical trial network group in the United States (US) [4]. This framework spans the three stages of “Concept Generation and Development”, “Scientific Evaluation”, and “Prioritization” leading to trial applications, particularly at the US National Cancer Institute. In 2023, Schuster et al. published a guiding document that reflects on the meaning of “engagement”, along with two conceptualizations of this construct [5]. Drawing from prior works [6], the authors conceptualized engagement as a state involving individuals’ cognitive and emotional processes expressed as behaviours and a process that involves individuals’ transitioning between the stages of “becoming engaged”, “staying engaged”, “disengaging”, and “re-engaging”.

The increasing risk of cancer among adolescent and young adults (AYAs, ages 15–39 years) in many jurisdictions has led to the recognition of this important patient population [7,8,9]. Patient engagement in research is particularly relevant for AYAs due to unique age-related challenges (e.g., sexual and reproductive health) [10]. However, the reporting of experiences with patient engagement in research with the AYA cancer patient population is very limited. In 2022, van Ham et al. conducted semi-structured qualitative interviews with researchers (*n* = 9) and AYA cancer patients (*n* = 3) on experiences with patient engagement in research in Denmark [11]. The authors reported practical findings including an adjusted involvement matrix based on nine phases of research and five roles for patient research partners (PRPs). Implications include creating awareness of the value of patient engagement in research at the individual level, advocating for training AYAs and researchers in this respect, and at the institutional level, calling for structural changes to support this approach [11]. In 2023, Pappot reported experiences with patient engagement in research with a panel of 10–15 AYAs spanning multiple studies over 5 years at a cancer support facility in Finland [12]. The authors learned that AYA cancer patients are willing to use their cancer lived experience in research and other co-creation projects. To do so, they require the following: “developing a trusting environment”, “providing support”, “flexibility”, and “acknowledging tensions and imbalances”. Adding to this literature, our objective is to share lessons learned from conducting patient engagement research with AYA cancer patients which have led to the proposed framework. Accordingly, we aim to provide comprehensive guidance for actively engaging AYA cancer patients and emphasize their essential role in achieving meaningful outcomes, hence positioning them as integral members of the entirety of the research process.

## 2. Research Context

Our proposed framework for meaningful engagement with AYA cancer PRPs is based on lessons learned from the “Following reproductive and sexual health care and outcomes of adolescent and young adult cancer patients” (“FUTURE” Study), which is funded by the Canadian Institutes of Health Research (CIHR). In brief, the FUTURE Study uses qualitative methods (focus groups) to explore impacts of cancer diagnosis and treatment on the sexual and reproductive health of AYA cancer patients in Canada. Since its inception, the study has prioritized patient engagement through the four guiding principles of the CIHR Strategy for Patient-Orientated Research Patient Engagement Framework: inclusiveness, support, mutual respect, and co-building [13]. The FUTURE Study team includes four PRPs and one researcher with lived experience in AYA cancer, who have been involved throughout the study in various roles and capacities.

**Figure 1:**
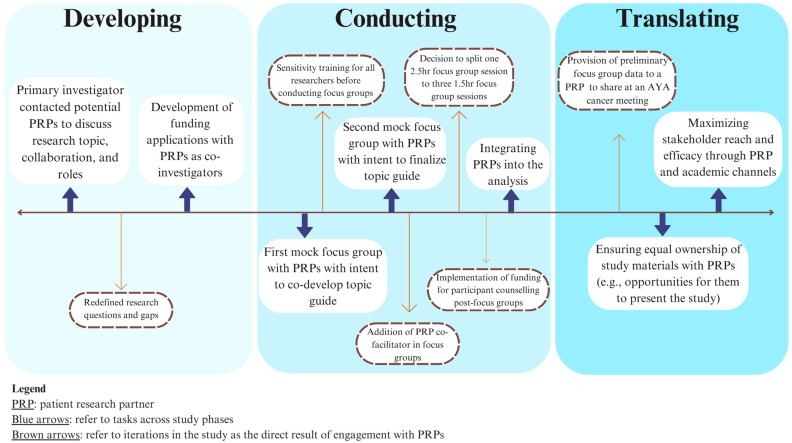
Overview of framework development through the FUTURE Study.

**Figure 2:**
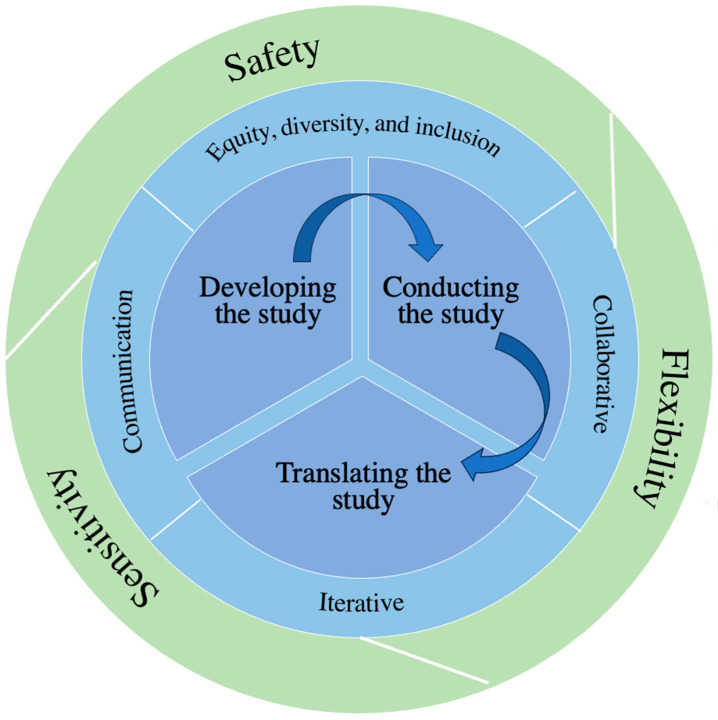
Overview of framework for patient engagement in qualitative research on adolescent and young adult cancer.

A 2020 scoping review by McMeekin et al. summarized the development of methodological frameworks and proposed a three-phase approach to their development [14]. We aligned with these three phases for the development of our proposed framework. For phase 1, identifying evidence to inform the methodological framework, aside from our experiences with the FUTURE Study, we drew from the aforementioned works by van Ham et al. and Pappot et al. on conducting patient engaged research with AYAs [11,12] to identify gaps and opportunities in AYA cancer patient engagement. Given the methodologic and content focus of the FUTURE Study, we also drew from Sherriff et al.’s 2014 work on considerations on conducting focus groups to evaluate sexual health service interventions for young people [15]. For phase 2, developing the methodological framework, we conducted an iterative engagement process throughout the FUTURE Study with the four PRPs and one researcher with lived experience in AYA cancer on our team to propose and refine the framework as the study progressed. In phase 3, evaluating and refining, we presented the framework to the FUTURE Study team—which includes an interdisciplinary team of four AYA cancer PRPs, researchers, and clinician/researchers—and integrated changes. While we propose this framework for qualitative research, aspects may be applicable to other research approaches. The framework is summarized in Table 1 and involves overarching principles which are tenets that apply across the entirety of the framework, considerations that apply across all phases of a research study, and tasks that apply to specific phases of a research study. Figure 1 provides an overview of the development of the framework through the FUTURE Study, and Figure 2 shows the relationships among all components of the framework. In describing the framework below, we provide examples from the FUTURE Study involving AYA cancer PRPs and integrate available evidence where applicable.

## 3. Framework

### 3.1. Overarching Principles

Three overarching principles guide our proposed framework. Throughout the framework, we repeatedly mention the importance of **safety** (ensuring that AYA cancer PRPs feel physically and emotionally safe throughout the research study), **flexibility** (providing space for the diverse needs and lifestyles of AYA cancer PRPs in their research study contribution), and **sensitivity** (approaching AYA cancer PRPs with empathy and awareness, while integrating moments of acknowledgement and support throughout the research process).

### 3.2. Considerations across All Study Phases

*Collaboration:* A fully collaborative approach involves PRPs from start to finish in a research study, that is, from developing to conducting and translating the study. This has been shown to increase meaningful engagement in AYA cancer patients [11], instead of one-off engagement or consultation with PRPs. Recognizing that research studies differ in terms of level and type of collaboration required, PRPs should be involved in the process of defining the nature and timing of the collaboration. Practically, this includes discussing what role(s) PRPs will have. Through discourse with researchers and AYA cancer patients, van Ham et al. found that AYA cancer PRPs can have dynamic roles, from providing practical support to being the lead of their own project [11]. The PRP roles described, including “listener” (the one who is given information), “co-thinker” (the one who is asked to give opinions), “advisor” (the one who gives [un]solicited advice), “partner” (the one who works as an equal partner), and “decision-maker” (the one who takes initiative or the [final] decision), are excellent starting points for discussions with PRPs on the role(s) they would like to take on for a research study [11]. The clear delineation of roles is a key facet of meaningful engagement, especially as PRPs may engage differently in various research studies [16]. Finally, this clear delineation of roles is also important given that AYA cancer PRPs have various commitments, including schooling, working, parenting, and navigating a complex disease.

*Iteration:* Having PRPs enhances a research study through their lived experience of disease, leading to unique perspectives that researchers do not have. Their inputs may lead to changes in the research study, introducing an iterative process, with changes being implemented during relevant phases, leading to overall improvement. This iterative nature aligns with the process of engagement described by Schuster et al., including active co-creation with patients in a dynamic and cyclical manner through periods of ”engagement”, ”disengagement”, and “re-engagement” [5]. To support this, one must consider and build in flexibility into the research study to allow for iteration in relevant phases. Examples of building in flexibility include allocating time prior to major milestones (e.g., grant deadline and manuscript submission) for PRPs to review the study material. It is also important to recognize when it is feasible to implement changes and when it is not. For example, following piloting data collection methods (i.e., focus groups) for the FUTURE Study, our PRP (H.B.) provided input on having a patient co-facilitate focus groups as a means to build trust and provide support for participants. As this was early in the study, it was feasible to implement this change in future data collection, including allowing for time for facilitation training prior to data collection. In addition to flexibility, we also recommend creating opportunities for conversations with PRPs on inputs and need for iterations within the research study. These conversations allow PRPs to brainstorm and to have opportunities for sharing life experiences relating to the study. In our experience in the FUTURE Study, the need for iterations was more often shared during these conversations as opposed to via email, as the former have fewer barriers. To ensure that opportunities for these conversations were retained, we built space for casual phone chats with PRPs as needed.

*Communication:* Communication among research study team members is important in a study, and with PRPs, principles of flexibility and safety must be additionally incorporated. Key considerations include which researcher(s) will communicate with the PRPs and what method of communication will be used. Establishing key contacts among research team members for PRPs helps with logistics and also builds trust and nurtures safety for PRPs. As PRPs tend to engage with research studies outside of their regular commitments, the hours in which they can meet and discuss the research study may be in the evenings or weekends [7]. Additionally, the preferred mode of communication may not always be email or online meetings, especially when discussions are urgent. This is important when working with an AYA cancer patient population, who often juggle many intersecting responsibilities that require flexibility. In the FUTURE Study, the principal investigator (M.D.V.) and PhD researcher/study coordinator (N.O.) are the main contacts for PRPs. We use three modes of communication—emails, telephone calls, and text messages. We find that phone calls are the most efficient way to share ideas so they are not forgotten, and text messages are used to relay quick information (e.g., not being able to make a meeting).

*Equity, diversity, and inclusion (EDI):* Disparities in cancer occurrence, treatment, and lifelong care are prevalent in the AYA cancer patient population [17] and AYA oncology trials [18,19]. More than ever, there have been calls for consideration of EDI in cancer research [20,21]. This, combined with the lack of representation, which has also been noted as a challenge in patient engagement in research [22,23] and oncology research [24], made it particularly important to address EDI in the FUTURE Study. Our consideration of EDI when working with PRPs included taking steps to ensure representation among PRPs, reflecting on internal biases, and implementing measures for PRPs to be supported and seen. With the focus of the FUTURE Study on AYA cancer patients’ sexual and reproductive health, representation of identities among PRPs recognizes the intersections of age, sex, gender, race, and ethnicity. Therefore, we aimed to include diverse identities when inviting PRPs, while refraining from identity tokenization. At the outset of the FUTURE Study, one of our PRPs (D.T.) facilitated a workshop wherein research team members were invited to reflect on their identities and how they intersect with their research. By doing so, they were able to identify biases and approaches to dismantling these prior to conducting the FUTURE Study. Finally, measures to ensure that PRPs are supported and seen are inextricably linked to the aforementioned considerations of collaboration and communication and also guided by tenets of safety, flexibility, and sensitivity. Providing emotional support to AYA cancer PRPs will ensure both their wellbeing and the success of the collaboration. Safety measures and support can be in the form of flexible timelines and expectations, establishing feedback mechanisms for PRPs to express their concerns, cultivating a supportive team through open communication and recognition of stressors, and access to mental health resources as needed.

### 3.3. Considerations for Specific Study Phases

#### 3.3.1. Developing the Study

This phase involves interrelated tasks—planning for engagement with PRPs, integrating PRPs’ perspectives, and funding PRPs. In the FUTURE Study, these tasks were performed in a concurrent, overlapping, and iterative manner.

*Planning for engagement with PRPs*: In the FUTURE Study, we engaged with potential PRPs through existing research networks. The process involved the primary investigator (PI) reaching out to the potential PRP, setting up an introductory meeting, discussing the potential research topic at hand, outlining phases of the research, providing examples of PRP roles from prior research, and establishing the level of interest in collaboration. Importantly, this was an exploratory process which also involved establishing preliminary communication expectations, providing space for potential PRPs to ask follow-up questions, and allowing them to have time to make the decision on their involvement. In this phase, we were aware of how many PRPs we were looking for and only reached out when we had the capacity for more PRPs on the team.

*Integrating PRPs’ perspectives:* In the FUTURE Study, we seek to address the research gap on sexual and reproductive health experiences and outcomes among AYA cancer patients. As we confirmed PRPs’ involvement with the research team, we revisited this research gap to seek their perspectives, which we then integrated into the developing the research proposal/protocol. How a research study integrates PRPs’ lived experiences and knowledge emphasizes the importance of having an iterative framework for PRP engagement. Considerations include what are PRPs’ experiences of the topic of the research study, what have they seen in their communities on this topic, and what are the questions they would like to ask about this topic? Addressing these considerations and applying relevant changes to the research study—research question, proposal, and protocol—will increase legitimacy, and the results can be better translated to the community [16]. Indeed, through the sharing of their unique perspectives and experiences with the research team, PRPs can help identify previously overlooked or underrepresented aspects of the research topic. For instance, in the FUTURE Study, there were opportunities for PRPs in the design phase, including participating in initial meetings and piloting research tasks (e.g., data collection methods). Their involvement helped us refine the study objectives by highlighting relevant aspects of sexual and reproductive health experiences among AYA cancer patients. The input provided by PRPs not only improved the depth of information that would be collected but also enhanced the study’s patient-centered approach. By actively involving PRPs and adapting the research to capture their insights, researchers can refine and bridge the identified research gaps effectively [25]. This collaborative approach enhances the patient-oriented relevance of research, ultimately leading to more meaningful and impactful outcomes [25].

*Funding PRPs*: When conducting patient engagement in research, funding is both a task (e.g., seeking funding) and a consideration (e.g., honorariums for PRPs). As PRPs may not routinely be involved in applying for funding, this may be an intimidating process. In the FUTURE Study, the acknowledgement and/or invitation of PRPs as co-investigators/collaborators on our research funding applications was an integral aspect of demonstrating respect and providing ownership to PRPs on their contribution to the study. By including PRPs as co-investigators/collaborators on our funding applications, we created an environment that encourages valuable feedback while also promoting their active roles within the research team. Although ownership is important, the process of submitting research funding applications is generally challenging and inaccessible for the lay person and has many barriers in place that prevent active engagement. To overcome these barriers, our research team facilitated the funding application process by providing detailed templates for letters of support, sending reminders of grant deadlines and requests, as well as assisting in the development of CVs and any other documents that were requested from PRPs.

As a consideration, the acknowledgment of PRPs’ contributions through honorariums must be integrated into research budgets. Honorarium rates should reflect not only the emotional burden associated with their collaboration but also their time and lived expertise. A 2023 systematic review on recognition of patient partner contributions to health research found that 91% of the eligible studies offered a type of compensation to patient partners. However, more than half of these compensations were non-financial (e.g., acknowledgment on research outputs and co-authorship). Barriers such as lack of funding and absence of institutional policy/guidance were noted, while enablers were consideration of compensation when developing the budget and adequate project funding [26]. To overcome these barriers, there are recommendations provided by research bodies and/or non-profit organizations, such as the BC SUPPORT (Support for People & Patient-Oriented Research & Trials) Unit [27]. Generally, erring on the side of higher pay is highly recommended, as Hamilton et al. noted that this compensation is a means of demonstrating appropriate respect and recognition of the valuable role that PRPs play in a research study [28]. In our experience in the FUTURE Study, we opted for higher honorarium rates. We also employed one-time payments to PRPs to streamline the process and minimize administrative burdens, such as tracking and submitting hours.

#### 3.3.2. Conducting the Study

This phase involves sequential tasks that pertain to conducting a research study. In the FUTURE Study, these included developing study materials, which we have completed, and recruiting participants, collecting data, and analyzing data, which are ongoing. Although sequential, we note the importance of an iterative process and allowing for flexibility to incorporate changes in relevant tasks with PRPs’ inputs.

*Developing study materials:* With respect to the development of study materials, co-creation with PRPs has been shown to increase enrolment in studies/reduce attrition, improve data collection tools, and increase knowledge translation (KT) penetration/efficacy [16,29,30,31]. We found that co-creation with PRPs also leads to iterations of the material. For example, as part of the study development process, we conducted two mock focus groups with our PRPs to test the topic guide. PRPs not only provided feedback on the topic guide; they also provided suggestions, particularly additional questions that are relevant to the lived experiences of AYA cancer patients. Beyond co-creating, we also challenge researchers to consider how PRPs may lead aspects of the research study. The mock focus groups with PRPs in our study further emphasized sensitivities around AYA cancer patients’ experiences with sexual and reproductive health and established the need to foster safety within future focus groups involving recruited participants, some of whom may not have experiences with research participation. A PRP (D.T.) suggested and led training for facilitating peer support groups such that we can apply approaches to subsequent focus groups to ensure safety and build a community. Guided by the principle of flexibility, we were able to integrate PRPs’ suggestions, with iterations within the research study leading to increased efficacy, higher-quality data, and greater safety for our participants.

*Recruiting participants:* AYA cancer patients have been historically underrepresented in research [32], and recruitment has been identified as a major challenge in this patient population [7,33]. Although AYAs are generally adept in social media, their busy schedules and lack of access to appropriate studies generally make them a hard population to reach [7]. Stemming from the development of study materials, the co-creation of recruitment strategies (e.g., channels to use) and materials (e.g., advertisement) with PRPs can facilitate accessibility, availability, and appropriateness. Indeed, materials used for research recruitment have often been placed in areas that are either inaccessible to AYA cancer patients (e.g., strict eligibility criteria that does not cover the entirety of the AYA age range and complex recruitment processes), unavailable (e.g., only adult cancer treatment centers, although AYAs are also present in pediatric centers), or inappropriate (e.g., only using in-person recruitment strategies, when AYA cancer patients mainly seek information online) [7]. In the FUTURE Study, PRPs were involved in the recruitment process. Through their communities, PRPs were able to suggest recruitment channels that we were not aware of (e.g., online forums and patient groups), share advertisement on these channels, and conduct shoulder tapping to invite people to participate or ask for help with sharing our study (e.g., online influencers). This was especially useful when recruiting cis-men, people of colour, gender- and sexual-orientation-diverse individuals, and other groups that generally have lower rates of participation in studies [34]. Through PRPs’ involvement in recruitment, we managed to overcome these barriers and exceed our ambitious diversity goals for recruitment.

*Collecting data:* Data collection in the FUTURE Study represents a study strength that would not have been possible without PRPs’ input and involvement. Through our iterative process, a PRP (H.B.) suggested the inclusion of a PRP co-facilitator in the focus groups, as they noted that this might make AYA cancer patients feel more at ease while discussing their sexual and reproductive health. This resulted in the inclusion of a patient co-facilitator (D.T., H.B., or M.D.V.) in all our focus groups. The patient co-facilitator and the research team member (N.O.) facilitated the focus groups equally. Anecdotally, we observed that participants were much more comfortable discussing their sexual and reproductive health in this setting, as they were able to relate to the stories shared by the patient co-facilitator. A PRP (D.T.) further suggested to expand participation from one 2.5 h focus group session to three 1.5 h focus group sessions. By doing so, we built a community within the cohort of participants who underwent the three sessions together and took our time with sensitive content. Another important suggestion from a PRP (D.T.) was providing support to participants beyond the focus groups, given the sensitivity of the topics discussed. The resultant iteration in the FUTURE Study was that participants were provided two counselling sessions with a counselor specializing in AYA cancer and reproductive/sexual health or reimbursement for two sessions with a counsellor they already worked with.

*Analyzing data:* Data analysis in qualitative research is a time-intensive process (involving initially coding transcripts, recoding, creating categories and themes, and drawing meaning from data) and is completed iteratively as data are collected. In prior qualitative research studies with PRPs, we learned that involving PRPs in the analysis process can be nuanced due to the jargon and level of expertise required to conduct qualitative analysis. Given these lessons, along with being mindful of the intensive nature of data analysis and a desire not to overburden PRPs in the FUTURE Study, we developed a structured process for integrating PRPs’ input into the analysis. This process involves (1) providing transcripts to PRPs, so that they gain insight into the focus groups that they may not have co-facilitated and see the range of data across sessions, (2) sharing the initial coding framework with PRPs for their insights and inputs, and (3) confirming final themes and subthemes with PRPs. At time of writing, we have only begun preliminary coding and have not fully implemented this process with PRPs yet. As with aforementioned phases and tasks, we anticipate receiving inputs from PRPs that may change this intended approach.

#### 3.3.3. Translating the Study

The KT phase represents the sharing of knowledge from the study. This phase may occur throughout the study, with an integrated KT approach, or at the end, with an end-of-grant KT approach [35,36]. Providing ownership to PRPs for study dissemination is integral to the development of trust and continued working relationship. In the FUTURE Study, PRPs are named as co-investigators on grant applications and co-authors on publications (with minimum one PRP as the second or third author). PRPs are also supported in their professional development; for example, we provided a PRP (D.T.) with preliminary focus group data to share in a presentation in an AYA cancer meeting. However, we caution that ownership may differ for PRPs, as some may wish to remain anonymous in their contribution. Finally, a major significance of PRPs is their integration with the AYA cancer community. As Manafo et al. indicated in their review [16], the translation of knowledge to the community is important for the empowerment of AYA cancer patients for self-advocacy. Through PRPs, we have been able to share the FUTURE Study with a wider audience, including the local, provincial, and national AYA cancer communities and advocacy groups that we would not otherwise have access to. For example, we gained access to AYA cancer social media groups that were only available through membership to the group. By fostering these connections through PRPs’ involvement, we were able to increase our capacity to reach various stakeholders who play pivotal roles in shaping the landscape of AYA cancer and sexual and reproductive health.

## 4. Summary

This paper introduces a framework for fostering meaningful engagement with AYA cancer PRPs, drawing insights from the “FUTURE” Study on reproductive and sexual health in AYA cancer. Guided by principles of safety, flexibility, and sensitivity, this framework covers the three major phases of a study (developing, conducting, and translating), while emphasizing collaboration; iteration; communication; and EDI. By transcending the role of a supplementary element, this framework positions patient engagement as an integral component for conducting impactful AYA cancer research, thereby enhancing its potential impact on patients.

## 5. Future Directions

In their scoping review on patient engagement in Canada, Manafo et al. found that when patients are engaged in research, there are improved research effectiveness and understanding of the community as well as increased enrolment and mobilisation of findings [16]. Patient engagement in cancer research has increased greatly over the last decade across many jurisdictions [3,5,12] and is of particular relevance for AYAs with lived experience of unique age-related challenges (e.g., sexual and reproductive health) [1,2,11,12].

This framework is meant to provide guidance for AYA cancer research patient engagement, specifically in qualitative research. By sharing lessons learned from the FUTURE Study, we hope to move the field from *passive* to *active* engagement, where patient engagement is no longer a “bonus” on a study but rather an integral component to achieving meaningful results. In our own research, we hope to navigate away from the idea of engagement with AYA cancer PRPs for their “lay expertise”, but rather provide PRPs with the research skills and knowledge to integrate their lifelong learning with research expertise as collaborators on research studies [37]. It is important to assess the validity and rigor of the proposed framework. To do so, future work can collect input regarding the feasibility of the framework from AYA cancer patients and researchers, pilot-test with small studies, and evaluate change in meaningful PRP engagement through longitudinal assessment of patient engagement in studies that utilize the framework. As the literature on experiences with PRPs in AYA cancer research grows, we also anticipate an opportunity for a synthesis (e.g., systematic review) where findings may be used validate and/or further refine our framework.

To support efforts towards embedding patient engagement in AYA cancer research, we hope for targeted funding opportunities, and spaces and opportunities for sharing lessons and experiences for researchers and PRPs. Ultimately, meaningful patient engagement in research is an important step towards improving lifelong outcomes for AYA cancer patients.

## Figures and Tables

**Table 1:** Collaborative and iterative framework for meaningful patient engagement in adolescent and young adult (AYA) cancer qualitative research.

Overarching Principles		Description
Safety		Ensuring that AYA cancer PRPs feel physically and emotionally safe throughout the research process
Flexibility		Providing space for the diverse needs and lifestyles of AYA cancer PRPs in their contribution to the research study
Sensitivity		Approaching AYA cancer PRPs with empathy and awareness, while thoroughly integrating moments of acknowledgement and support systems
Study Phase	Consideration (c)/Task (t)	Points
Across all study phases	Collaboration (c)	- What does “collaboration” mean to the research team? - What is the role of PRPs in the research team/research study?
	Iteration (c)	- Can flexibility be built into the research study to allow for changes based on PRPs’ inputs? In which study phase(s) would this be feasible? - Are there opportunities for conversations on iterations within the research study?
	Communication (c)	- Who will be the researcher(s) communicating with PRP(s)? - What method of communication (e.g., email, text messages, or team management applications) will be used?
	Equity, diversity, and inclusion (c)	- What steps have the research team taken to ensure representation of PRPs? - Has there been reflection on and the unlearning of the research team’s internal biases? - Are there safety measures in place to ensure that PRPs feel supported and seen?
Developing the study	Planning for engagement with PRPs (t)	- How will PRPs be recruited? - How many PRPs will be part of the team and what is their level of involvement?
	Funding PRPs (c, t)	- Have PRPs been listed as research team members on grant applications and provided opportunities for feedback? - Are there systems in place to alleviate the burden of funding applications on PRPs (e.g., assistance for preparing letters of support and CV development, etc.)? - How will PRPs be compensated for their involvement (e.g., honorariums)?
	Integrating PRPs’ perspectives (t)	- How will the research study integrate PRPs’ experience and knowledge? - Are there opportunities for redefining gaps from PRPs’ input?
Conducting the study	Developing study materials (t)	- Are study materials co-created with PRPs? - Do PRPs have opportunities to lead aspects of the study?
	Recruiting participants (t)	- Are recruitment strategies co-created with PRPs? - Are PRPs involved in recruitment?
	Collecting data (t)	- How have PRPs taken an active role during data collection (e.g., co-facilitator, moderator, etc.)? - Are there opportunities for revising data collection protocols according to PRPs’ input?
	Analyzing data (t)	- How are PRPs involved in data analysis? - Are PRPs provided with support (e.g., with research jargon and training on methods) to facilitate involvement in data analysis?
Translating the study	Ownership of study materials (t)	- Are PRPs listed as co-investigators/co-authors on study material? - Are PRPs supported in study-related pursuits?
	Maximizing KT (t)	- How will KT integrate PRP experience and knowledge? - Are PRP networks leveraged for KT?

## Data Availability

No new data were created or analyzed in this study. Data sharing is not applicable to this article.
